# Cytokeratin‐positive follicular dendritic cell sarcoma: A mimic of carcinoma

**DOI:** 10.1002/jha2.344

**Published:** 2021-12-08

**Authors:** Elizabeth Ricks, Shimin Hu, Zhihong Hu

**Affiliations:** ^1^ Department of Pathology and Laboratory Medicine The University of Texas Health Science Center at Houston Houston Texas USA; ^2^ Department of Hematopathology The University of Texas MD Anderson Cancer Center Houston Texas USA

**Keywords:** dendritic cells, diagnostic haematology, haematopathology

**FIGURE 1:**
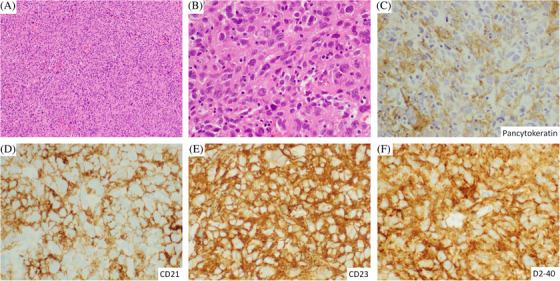
Cytokeratin‐positive follicular dendritic cell sarcoma. (A,B) H&E images show whorls of pleomorphic spindle cells with no discernable node architecture. (C) Pancytokeratin is focally positive. (D–F) CD21, CD23, and D2‐40 (podoplanin) are positive.

A 34‐year‐old male presented with night sweats, fatigue, and an enlarging left neck mass. Computed tomography (CT) scan showed a 5.6 × 4.2 × 3.7 cm necrotic mass in the left upper lateral carotid space. Histologic examination of an incisional biopsy of the mass demonstrated pleomorphic, plump spindle cells forming whorls with no discernable node architecture (Figure 1, panels A and B). The tumour cells were large with oval nuclei, vesicular chromatin, distinct nucleoli, and a moderate amount of eosinophilic cytoplasm. Occasional binucleated and multinucleated tumour cells as well as rare tumour cells with nuclear pseudoinclusions and atypical mitotic figures were present. Scattered small mature lymphocytes were seen. Focal necrosis was also noted. By immunohistochemistry, the tumour cells were positive for pancytokeratin (focal) (Figure 1, panel C), CD21, CD23, CD35, D2‐40 (podoplanin), and clusterin (Figure 1, panels D–F), and negative for CD3, CD20, CD30, CD45, ALK‐1, EMA, PAX5, PDL1, and S100 stains. The patient was diagnosed with follicular dendritic cell (FDC) sarcoma.

FDC sarcoma rarely occurs as the solitary mass in the head and neck region. The unusual focal positivity for pancytokeratin highlights the importance of comprehensive immunohistochemistry to confirm the diagnosis of FDC sarcoma since this case is positive for pancytokeratin, mimicking other neoplasms such as poorly differentiated carcinoma.

